# Dataset of ecosystem services in Beijing and its surrounding areas

**DOI:** 10.1016/j.dib.2020.105151

**Published:** 2020-01-17

**Authors:** Tianqian Chen, Zhe Feng, Huafu Zhao, Kening Wu

**Affiliations:** a School of Land Science and Technology, China University of Geosciences, Beijing 100083, China; b Key Laboratory of Land Consolidation and Rehabilitation, Ministry of Natural Resources, Beijing 100035, China

**Keywords:** Ecosystem service, NDVI, InVEST model, RUSLE, Daily carrying capacity, Beijing and its surrounding areas

## Abstract

This data article describes the multiple ecosystem services in Beijing and surrounding areas, including grain providing, water yield, carbon sequestration, soil retention, purified water service, cultural services, and habitat quality. These data are mainly from public data sets such as the Harmonized World Soil Database. These data can be used to improve the optimization of human well-being in the social-ecological system and further achieve regional sustainable development.

**Table:** Specifications Table

Subject	Ecology
Specific subject area	Ecosystem services
Type of data	Table Figure Raster (Geotiff)
How data were acquired	The raw data can be downloaded from some public datasets or from the supplementary data files. Public datasets: NDVI (DOI:10.12078/2018060601); Carbon density in Chinese terrestrial ecosystems (DOI: 10.11922/sciencedb.603); Evapotranspiration (MODIS/Terra Net Evapotranspiration Gap-Filled 8-Day L4 Global 500m SIN Grid V006, https://e4ftl01.cr.usgs.gov/MOLT/MOD16A2GF.006/, LP DAAC Data Pool provides direct access to available products via HTTPS); DEM (http://srtm.csi.cgiar.org/srtmdata/); Harmonized World Soil Database v1.2 (http://www.fao.org/soils-portal/soil-survey/soil-maps-and-databases/harmonized-world-soil-database-v12/en/); Rainfall (http://www.geodata.cn); Land use and land cover (http://www.resdc.cn/data.aspx?DATAID=283) Supplementary data files: Raster files of eleven ecosystem services (ESdata.zip); Biophysical table of six land use and land cover type (biophysical.xlsx); Grain production in Beijing and surrounding areas (grain.xlsx).
Data format	Raw and analyzed
Parameters for data collection	Ten ecosystem services in Beijing and its surrounding area, including grain providing, water yield, carbon sequestration, soil retention, purified water service (N export; P export), cultural services (natural landscape; history culture; entertainment), and habitat quality.
Description of data collection	The raw data, including spatial data and statistical data, is mainly downloaded from some public datasets, such as NASA earth science data (https://earthdata.nasa.gov/), CGIAR (http://srtm.csi.cgiar.org/srtmdata/) and Harmonized World Soil Database. The ecosystem service dataset is derived from the analysis and processing of the raw data.
Data source location	Beijing and its surrounding areas, including two municipalities (e.g., Beijing and Tianjin) and five prefecture-level cities (e.g., Hebei Province, Zhangjiakou, Baoding, Langfang, Tangshan, and Chengde).
Data accessibility	Data is available within this article in the link provided. Some data can be downloaded from the attachments, including “ES_Beijing_and_Surrounding.zip” and “rawdata.zip”.
Related research article	Chen T Q, Feng Z, Zhao H F, et al. Identification of Ecosystem Service Bundles and Driving Factors in Beijing and its Surrounding Areas. Science of the Total Environment. In Press [1].

**Table:** 

**Value of the Data** • The dataset helps to understand that ecosystem functions are reflected in human society in the form of ecosystem services. • Researchers can use data for ES assessment. • Decision makers can improve management practices based on ecosystem services. • Data can be used to further insight into the trade-offs and synergies and identify ecosystem service bundles and driving factors. • This dataset helps to understand the ecosystem service space configuration in high-intensity human activity areas.

## 1 Data description

The dataset contains spatial data for multiple ecosystem services in Beijing and surrounding areas. Ten ES were selected for valuing and mapping, including grain providing (GP), water yield (WY), carbon sequestration (CS), soil retention (SEC), purified water service, cultural services, and habitat quality (HQ). Spatialized data is used for the identification of ecosystem service bundles and driving factors. The visual representation and the files of these services can be downloaded from supplementary data files (“ESdata.zip”). The spatial resolution of ES is 1 km × 1 km, and China Lambert Conformal Conic is the projection coordinate system. In addition, the raw data for ecosystem services mapping is can be download in from supplementary data files (“Rawdata.zip ") or displayed directly in the table.

Part of the raw data as shown in the table. FAO table (http://www.fao.org/docrep/X0490E/x0490e0b.htm) is used to calculate evapotranspiration coefficient (kc), which uses average monthly reference evapotranspiration (PET) (https://earthdata.nasa.gov/) (Table 1). Z is an empirical constant, as shown in Table 2. Based on existing research, the number of rain days (http://data.cma.cn/) is used to calculate the Z parameter [4]. The dataset of carbon density in Chinese terrestrial ecosystems (http://www.cnern.org.cn/) is used to calculate carbon density data for six land use and land cover type (Table 3).

**Table 1:** The monthly PET and kc.

Month	average PET (mm/month)	Month	average PET (mm/month)	Land use and land cover	kc
JAN	39.34	JUL	175.93	Cropland	0.57
FEB	59.57	AUG	140.68	Woodland	0.90
MAR	125.30	SEP	109.65	Grassland	0.85
APR	193.53	OCT	112.74	Surface waters	0.72
MAY	238.46	NOV	43.29	Built-up land	0.30
JUN	185.72	DEC	32.76	Undeveloped land	0.50

**Table 2:** The monthly rainy days and Z parameter.

Month	1	2	3	4	5	6	Z parameter

Number of stations	440.00	484.13	537.00	535.81	538.00	535.81	
Number of rain days	2.03	3.13	1.65	4.26	5.00	6.11	

Month	7	8	9	10	11	12	11.04

Number of stations	538.00	536.94	520.65	538.00	519.68	537.00	
Number of rain days	6.04	6.06	7.44	4.41	7.20	1.85

**Table 3:** Carbon density of each land use type.

Land use and land cover	C_above	C_below	C_soil	C_dead
Cropland	15.8	47.83	64.5	9.82
Woodland	37.39	68.69	105.62	14.11
Grassland	30.7	51.27	92.77	10.55
Surface waters	8.2	39.5	0	0
Built-up land	1.2	27.6	61.71	0
Undeveloped land	7.23	32.4	78.48	0

## 2 Experimental design, materials, and methods

It's worth emphasizing that data is provided as a zipped folder under the name “ES_Beijing_and_Surrounding”. There's a description of how to calculate these ecosystem services from raw data analysis, including design, data acquisition, and methods.

**Fig. 1:**
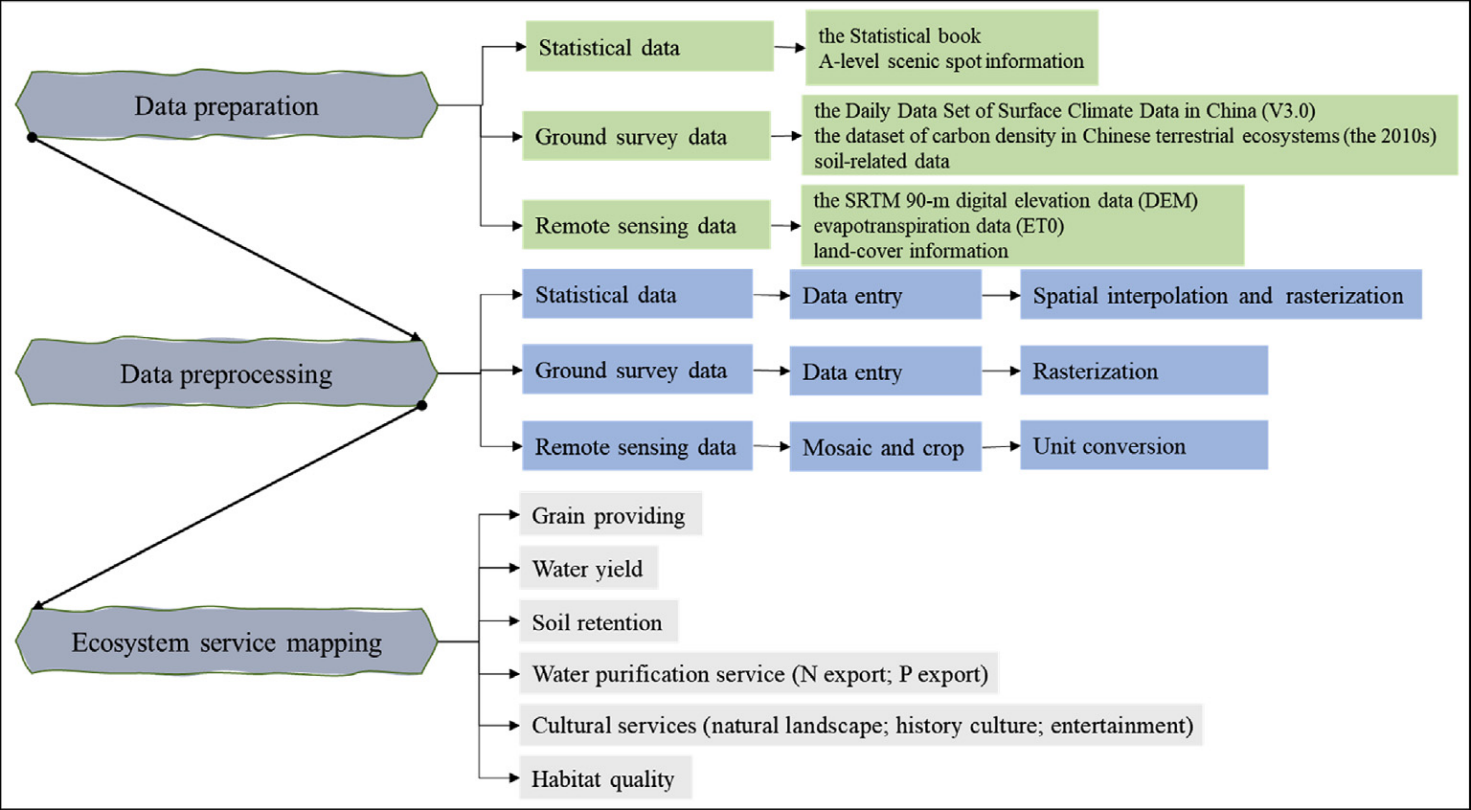
Dataset's production process of ecosystem services in Beijing and surrounding areas.

**Fig. 2:**
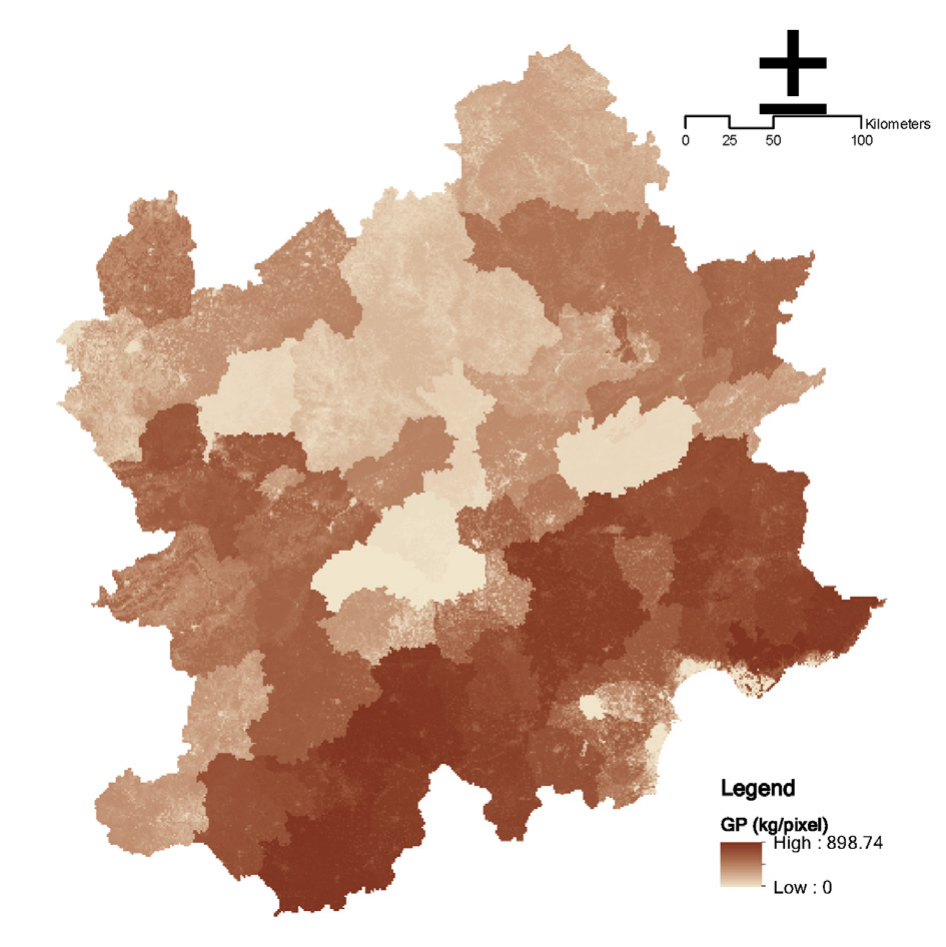
Grain providing data in the Beijing and its surrounding area.

**Fig. 3:**
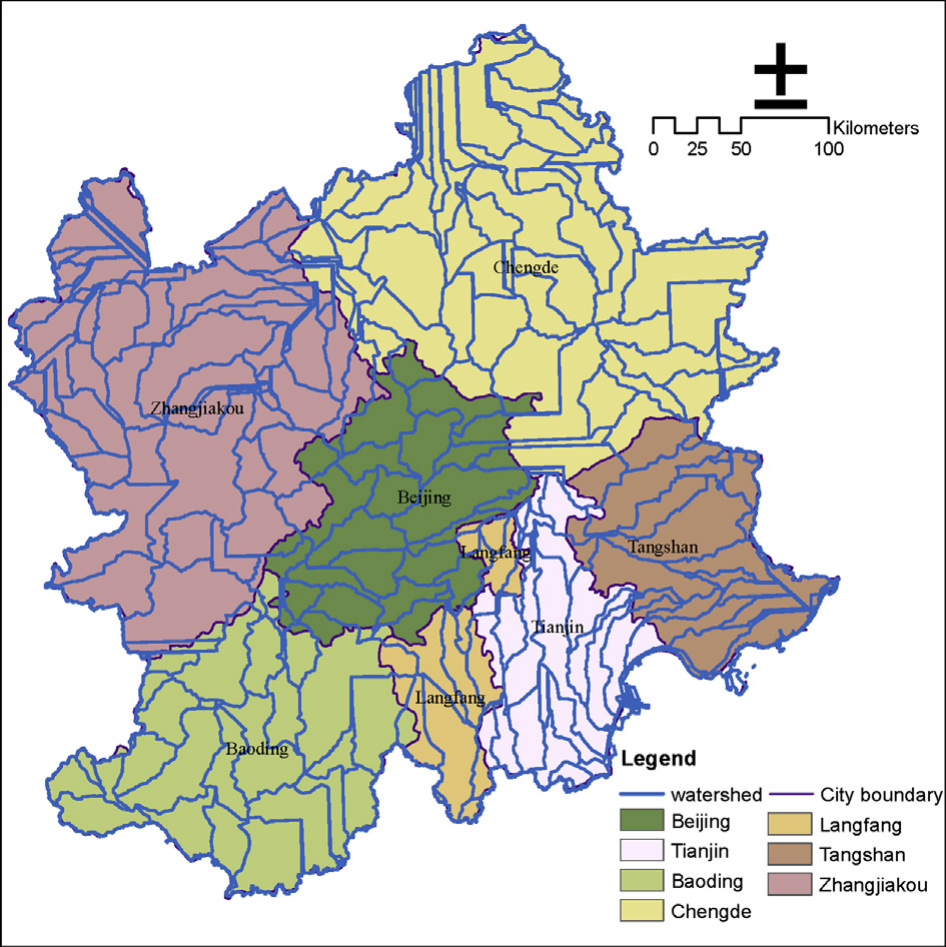
Watershed distribution in the Beijing and its surrounding area.

**Fig. 4:**
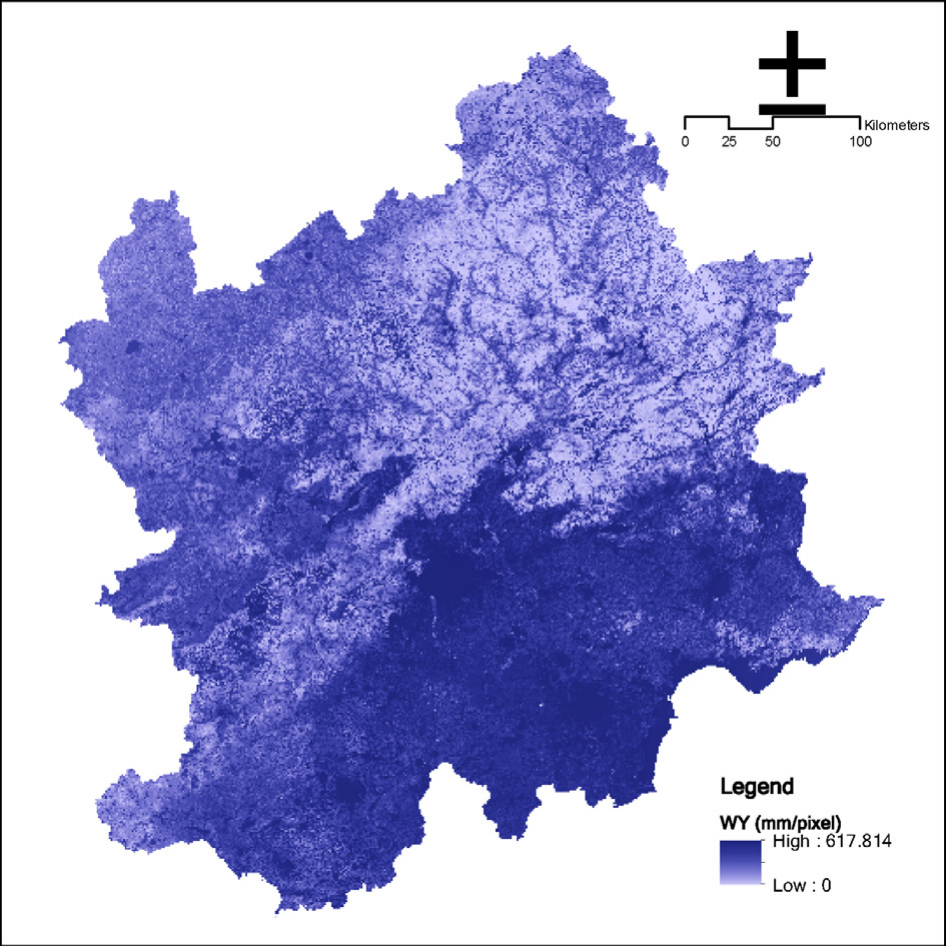
Annual water yield in the Beijing and its surrounding area.

The first step is the overall frame design, as shown in Fig. 1. Obtaining ecosystem service data in Beijing and surrounding areas is the goal, and the social status and ecological process characteristics of Beijing and surrounding areas are the basis. With reference to relevant research, ten ecosystem services were selected, including grain providing, water yield, carbon sequestration, soil retention, water purification service (N export; P export), cultural services (natural landscape; history culture; entertainment), and habitat quality (See Fig. 2, Fig. 3, Fig. 4).

The second step is to get the data. Some public data is available for download. The download address is available within this article in the link provided.

The third step is to select the appropriate model and tools to map these ecosystem services. The data and tools used for each service are shown in Table 4.

**Table 4:** Detailed information on ecosystem service mapping (including ecosystem service type and method).

Ecosystem service	Data required	Method	Unit
Grain providing	Grain production and NDVI	Map algebra	t/pixel
Water yield	DEM, rainfall, evapotranspiration, soil data, and LUCC	InVEST < -Annual Water Yield	mm/pixel
Carbon sequestration	Carbon density and LUCC	InVEST < -Carbon Storage and Sequestration	Mg/pixel
Soil retention	soil data, rainfall erosion factor, DEM, and NDVI	Map algebra	10 t/pixel
Water purification service (N export; P export)	DEM, LUCC, rainfall, and biophysical data	InVEST < -Nutrient Delivery Ratio	kg/pixel
Cultural services (natural landscape; history culture; entertainment)	Maximum daily carrying capacity in scenic areas	Inverse distance weight interpolation	10,000 people
Habitat quality	LUCC, biophysical data	InVEST < Habitat Quality	ratio

### 2.1 Grain providing

The grain production data is the grain, vegetable, and fruit supply data of the districts and counties, and is rasterized based on the foundation of the NDVI.

(1) $${\text{GP}}_{\text{ij}}=\frac{{\text{NDVI}}_{\text{ij}}}{{\text{NDVI}}_{\text{i}}}{\text{GP}}_{\text{i}}$$

where ${\text{GP}}_{\text{ij}}$ is the jth pixel of GP in the ith county, ${\text{NDVI}}_{\text{ij}}$ is the jth pixel of NDVI in the ith county, ${\text{NDVI}}_{\text{i}}$ is the NDVI in the ith county, and ${\text{GP}}_{\text{ij}}$ is the GP in the ith county.

### 2.2 Water yield

The InVEST annual water yield model is applied to estimate the total and average volume of water of each sub-basin in the research area. The model is based on the Budyko curve and the annual average precipitation.

The model requires some important parameters, including sub-watershed, evapotranspiration, land use and land cover, root depth, evaporation coefficient, empirical constant Z, etc.

(1) The sub-watershed is generated by DEM through the hydrologic analytical toolset of ArcGIS.
(2) Evapotranspiration data stems from NASA public datasets. The conversion factor (λ = 2.45MJ/kg) is used to convert the latent heat flux to evapotranspiration in mm.
(3) Root depth originates from HWSD Formula 2 shows the calculation method of water content of plants with the adoption of the international classification standard of soil texture [2].

(2) $$\text{PAWC}=54.509-0.132\times \text{SAND}-0.003\times {\text{SAND }}^{2}-0.055\times \text{SILT}-0.006\;\times {\text{SILT}}^{2}-0.738\times \text{CLAY}+0.007\times {\text{CLAY}}^{2}-2.688\times \text{OM}+0.501\;\times {\text{OM}}^{2}$$

where PAWC is the water content available to plants, SAND is the percentage content of soil sand, SILT is the percentage content of soil silt, CLAY is the percentage content of soil clay, and OC is the percentage content of soil organic matter.

(4) LUCC includes six types, namely, crop, forest, grass, developed land, water, and undeveloped land.
(5) The maximum root depth in the biophysical table is determined in consideration of the current studies [3].
(6) The plant transpiration and evaporation coefficient kc are computed using the kc calculator provided by FAO.
(7) The statistical analysis of daily rainfall data from 121 meteorological stations in the research area is analyzed to verify whether the annual rainfall event number is approximately 55.18, and Z is 11.04. This task is undertaken to calculate Z using Formula 3 [4].

(3) Z=0.2×N

where Z is the empirical constant related to the local precipitation model and hydrogeological characteristics with its value ranging from 1 to 30; and N is the number of annual rainfall events.

### 2.3 Carbon sequestration

**Fig. 5:**
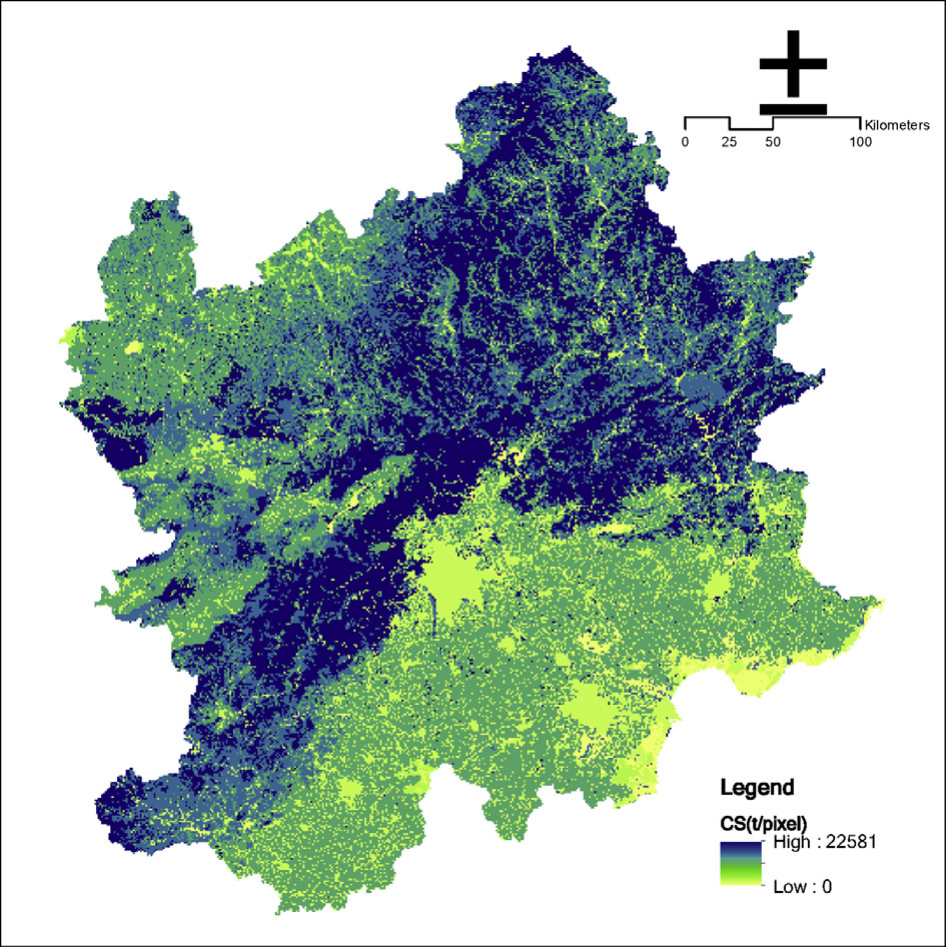
Carbon sequestration in the Beijing and its surrounding area.

**Fig. 6:**
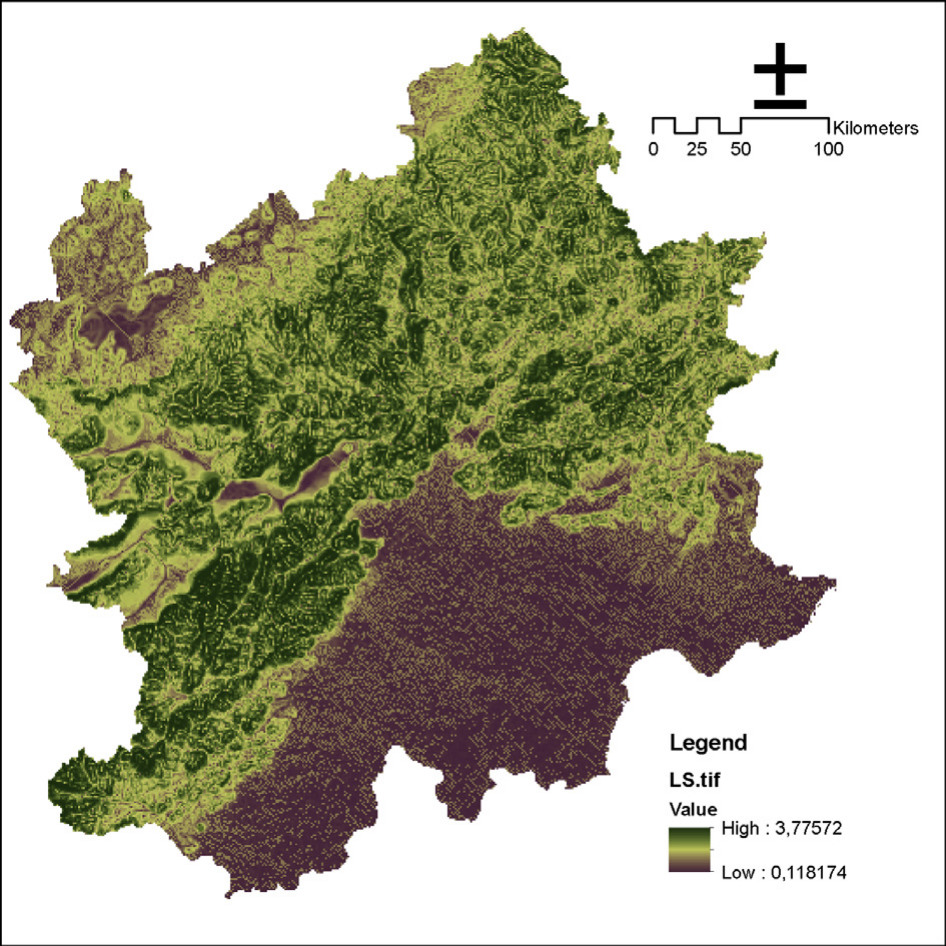
Topographic factor of the Beijing and its surrounding area.

**Fig. 7:**
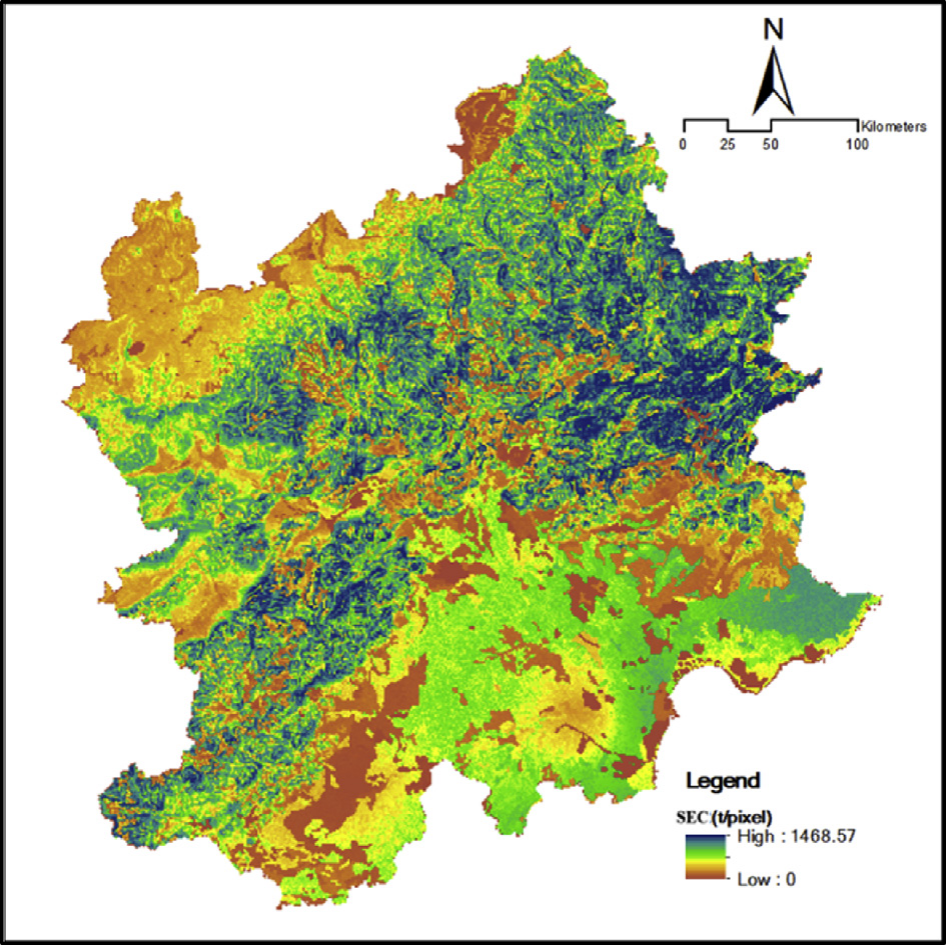
Spatial distribution of soil conservation services in the Beijing and its surrounding area.

**Fig. 8:**
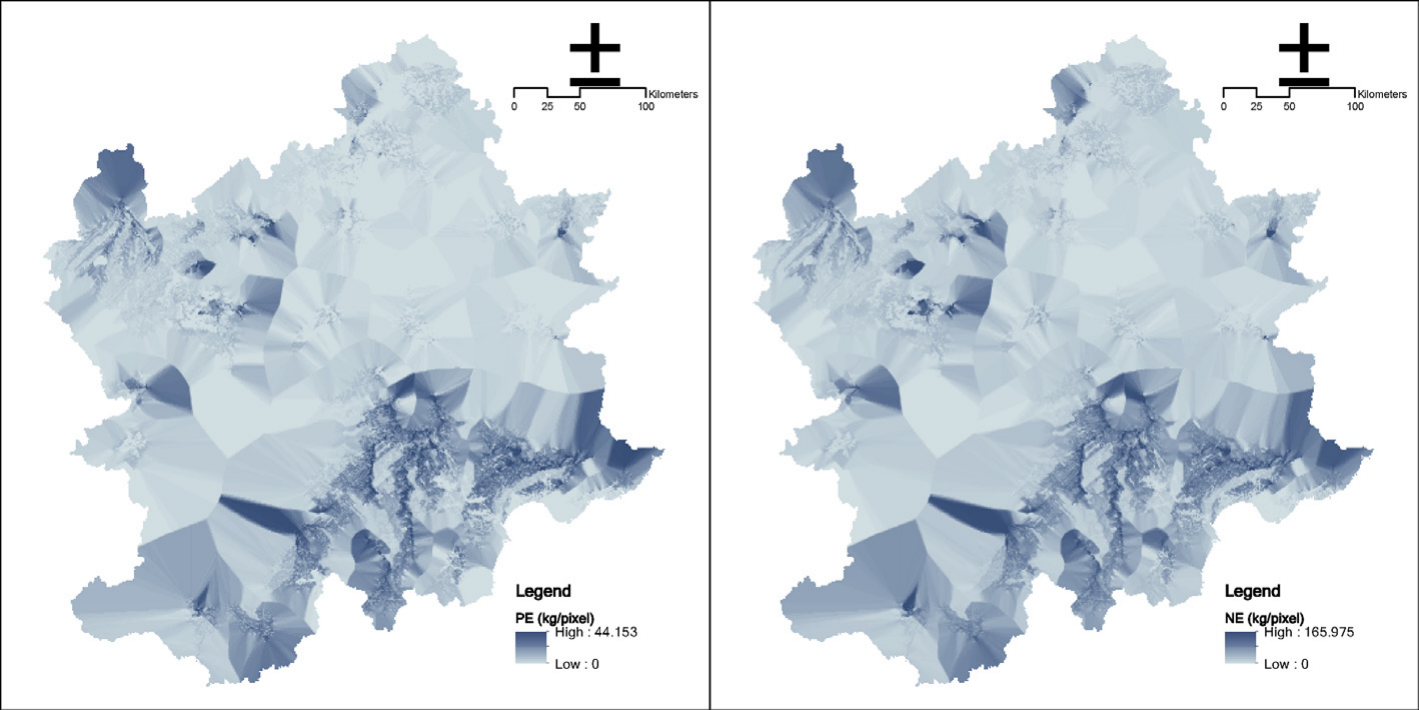
Water purification service in the Beijing and its surrounding area (the left one is N export, and the right one is P export).

The InVEST carbon storage and sequestration model is used to calculate the total amount of CS in the light of the corresponding carbon density and land use and land cover. The model can be simply expressed as the sum of the four carbon pools of aboveground biomass, belowground biomass, soil, and dead organic matter using Formula 4 (See Fig. 5, Fig. 6, Fig. 7, Fig. 8).

(4) $$\text{CS}=\overset{5}{\underset{\text{i}=1}{\sum }}{\text{S}}_{\text{i}}\times \left(\text{C}\_{\text{above}}_{\text{i}}+\text{C}\_{\text{below}}_{\text{i}}+\text{C}\_{\text{soil}}_{\text{i}}+\text{C}\_{\text{dead}}_{\text{i}}\right)$$

where i is the code of LUCC; and $\text{C}\_{\text{above}}_{\text{i}}$ is carbon density in aboveground biomass (megagrams/hectare); and $\text{C}\_{\text{below}}_{\text{i}}$ is carbon density in belowground biomass (megagrams/hectare); $\text{C}\_{\text{soil}}_{\text{i}}$ is carbon density in soil (megagrams/hectare); and $\text{C}\_{\text{dead}}_{\text{i}}$ is carbon density in dead matter (megagrams/hectare).

The main parameters:

(1) Carbon density is essential for this model. The “2010s China terrestrial ecosystem carbon density data set” is taken for reference to determine the carbon density data [5]. This data can be found in Table 3.
(2) Land use and land cover can be download from (http://www.resdc.cn/DataList.aspx).

### 2.4 Soil retention

SEC is quantified by the universal soil loss equation [6]. With the prediction of the annual amount of soil erosion, the difference between the results of the amount of the potential soil erosion and the actual amount of erosion is the quantity of soil conservation.

(5) $${\text{A}}_{\text{C}}=\text{R}\times \text{K}\times \text{LS}\times \left(1-\text{C}\times \text{P}\right)$$

where ${\text{A}}_{\text{C}}$ is the amount of SEC (t/(ha/yr)), R is the rainfall erosion index (MJ/mm/(ha/h/a)), K is the soil erosion factor, LS is the slope length-gradient factor, C is the crop/vegetation and management factor, and P is the support practice factor.

Various factors are involved, including rainfall patterns (R), soil type (K), terrain (LS), crop system(C), and management practice(P).

(1) R comes from the National Earth System Science Data Sharing Platform (http://www.geodata.cn/).
(2) With the application of the international classification standard of soil texture and the percentage content of soil sand, silt, and clay, K can be computed in light of Formula 8 by utilizing the EPIC model proposed by Williams and Arnold [7].

(6) $$\text{K}=\left(0.2+0.3\;\exp\left(-0.0256\times \text{SAND}\times \left(1-\frac{\text{SILT}}{100}\right)\right)\right)\times {\left(\frac{\text{SILT}}{\text{CLAY}+\text{SILT}}\right)}^{0.3}\times \left(1-\frac{0.25\times \text{OM}}{\text{OM}+\text{exp}\left(3.72-2.95\times \text{OM}\right)}\right)\times \left(1-\frac{0.7\times \left(1-\text{SAND}/100\right)}{\left(1-\frac{\text{SNAD}}{100}\right)+\text{exp}\left(-5.51+22.9\times \left(1-\text{SAND}/100\right)\right)}\right)$$

where SAND, SILT, CLAY, and OM are the percentages of sand, silt, clay, and organic carbon in the soil, respectively.

(3) LS can be acquired through terrain analysis of ArcGIS.

(4) C is acquired by utilizing the NDVI data, Cai et al.‘s [8] calculation method, and (7), (8).

(7) $$\text{f}=\frac{\left(\text{NDVI}-{\text{NDVI}}_{\text{min}}\right)}{\left({\text{NDVI}}_{\text{max}}-{\text{NDVI}}_{\text{min}}\right)}$$

(8) $$\text{C}=\{\begin{array}{cc} 1, & f=0\\ 0.6508-0.3436lgf, & 0<f\leq 78.3\%\\ 0, & f>78.3\% \end{array}$$

where ${\text{NDVI}}_{\text{min}}$ is the minimum of NDVI, ${\text{NDVI}}_{\text{max}}$ is the maximum of NDVI, f is the degree of vegetation coverage, and C is the crop/vegetation and management factor.

(5) The P factor was calculated through Wener method [9] (Formula 9).

(9) P=0.2+0.03α

where α is slope steepness (%), and P is the support practice factor.

### 2.5 Water purification service

N export and P export are typical representatives of water purification services. The InVEST nutrient delivery ratio model is used to calculate the regulating service.

The model calculates the pixel-level output data based on the nutrient load (load_i) and NDR of each pixel i and then merges into the total output of the basin range.

(10) $$\text{NDR}=\sum^{}{\text{load}}_{\text{i}}\times {\text{NDR}}_{\text{i}}\left({\text{D}}_{\text{up}},{\text{D}}_{\text{n}},{\text{eff}}_{\text{dn}}\right)$$

where NDR is nutrient delivery ratio (N export or P export); ${\text{load}}_{\text{i}}$ is nutrient load in pixel i; ${\text{D}}_{\text{up}}$ is a function of the upslope area; ${\text{D}}_{\text{n}}$ is a function of the downslope flow path; ${\text{eff}}_{\text{dn}}$ is retention efficiencies in pixel i.

The main parameters:

(1) The nutrient load is determined by referring to Harmel et al. [10] and Pärn et al. [11].
(2) The NatCap nutrition parameter database is used as a reference to determine retention efficiency.
(3) The maximum pixel is applied for retention lengths.

### 2.6 Cultural services

**Fig. 9:**
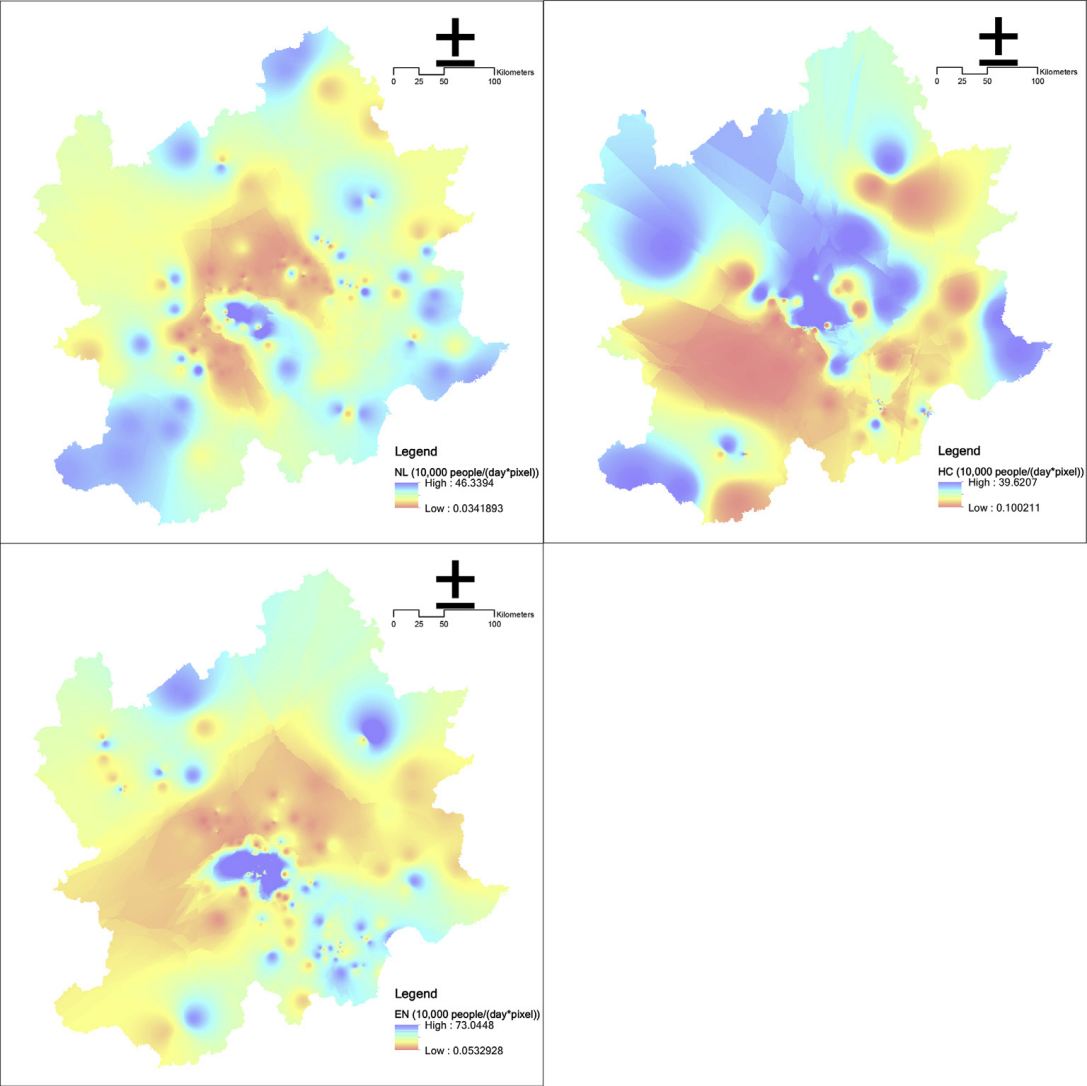
Cultural service in the Beijing and its surrounding area (NL means natural landscape; HC means history culture; EN means entertainment).

**Fig. 10:**
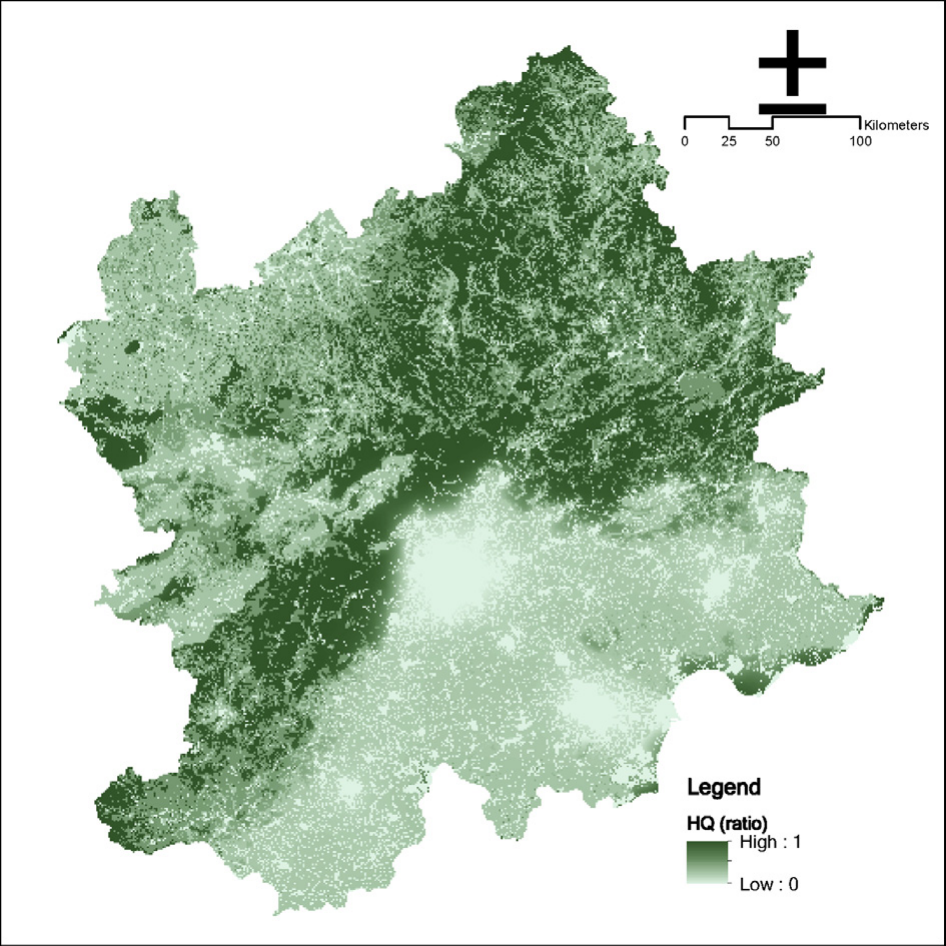
Habitat quality in the Beijing and its surrounding area.

The main index adopts the accounting data of the maximum daily carrying capacity of each A-level scenic spot implemented in China since 2014 to represent the cultural service and calculates through the inverse distance weight interpolation method. According to the service categories of scenic spots, cultural services can be divided into three types, including natural landscape, history culture, and entertainment (See Fig. 9, Fig. 10).

Some important parameters:

(1) The A-level scenic list was obtained from the Ministry of Culture and Tourism of the People's Republic of China.
(2) The longitude and latitude of the scenic spot are crawled through Baidu API.
(3) The maximum daily carrying capacity is obtained through government public data.

### 2.7 Habitat quality

The InVEST HQ model is selected to calculate HQ by combining the relevant information of land use and land cover and the diverse threat to ecology to constitute an HQ map.

Some important parameter:

(1) Current land use and land cover can be downloaded from public datasets.
(2) Threats data and sensitivity of land use and land cover types to each threat is based on existing research [12].
(4) Half-saturation constant is fixed into default value (0.5).
